# Peyer's Patches: The Immune Sensors of the Intestine

**DOI:** 10.4061/2010/823710

**Published:** 2010-09-19

**Authors:** Camille Jung, Jean-Pierre Hugot, Frédérick Barreau

**Affiliations:** ^1^UMR843 INSERM, Université Sorbonne Paris Cité-Diderot, Hôpital Robert Debré, 75019 Paris, France; ^2^Assistance Publique Hôpitaux de Paris, Hôpital Robert Debré, 75019 Paris, France

## Abstract

The gut-associated lymphoid tissue (GALT) consists of isolated or aggregated lymphoid follicles forming Peyer's patches (PPs). By their ability to transport luminal antigens and bacteria, PPs can be considered as the immune sensors of the intestine. PPs functions like induction of immune tolerance or defense against pathogens result from the complex interplay between immune cells located in the lymphoid follicles and the follicle-associated epithelium. This crosstalk seems to be regulated by pathogen recognition receptors, especially Nod2. Although TLR exerts a limited role in PP homeotasis, Nod2 regulates the number, size, and T-cell composition of PPs, in response to the gut flora. In turn, CD4^+^ T-cells present in the PP are able to modulate the paracellular and transcellular permeabilities. Two human disorders, Crohn's disease and graft-versus-host disease are thought to be driven by an abnormal response toward the commensal flora. They have been associated with NOD2 mutations and PP dysfunction.

## 1. Introduction

In the gut, discrimination between pathogens and commensal bacteria is achieved by the interaction of the intestinal epithelium with lymphoid cells. The gut-associated lymphoid tissue (GALT) consists of both isolated and aggregated lymphoid follicles [1] and is one of the largest lymphoid organs, containing up to 70% of the body's immunocytes. Aggregated lymphoid follicles were initially described by Marco Aurelio Severino in 1645 in Italy. They were named Peyer's Patches (PPs) after their detailed description by the Swiss pathologist Johann Conrad Peyer in 1677. PPs are composed by aggregated lymphoid follicles surrounded by a particular epithelium, the follicle-associated epithelium (FAE) that forms the interface between the GALT and the luminal microenvironment. The FAE contains specialized cells named M (for microfold) cells. These M-cells are able to transport luminal antigens and bacteria toward the underlying immune cells that activate or inhibit the immune response leading to either tolerance or systemic immune cell response. The aims of this paper are to describe the different actors and functions of the PP, their implication in the induction of immune tolerance and defense against pathogens and finally their role at the interface between innate and adaptive immunity.

## 2. Development, Architecture, and Functions of Peyer's Patches

**Figure 1:**
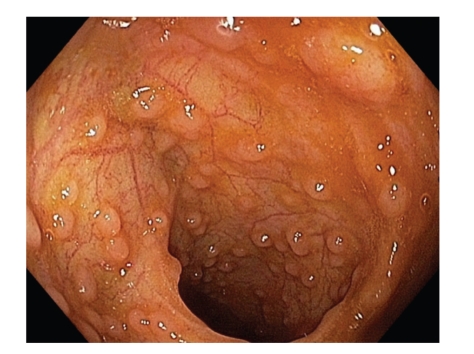
Peyer's patches in the distal ileum. PPs seen in a 20-years-old man during ileocolonoscopy. Note that PPs form a lymphoid ring in the distal ileum.

The postnatal development of PPs has been initially investigated by Cornes who reported in 1965 that the number of PPs peaks at ages 15–25 and then declines during the life [2]. Van Kruiningen et al. confirmed these findings [3] and noted that, in addition, the area occupied by PPs in the ileum is maximum in the third decade [4]. In the human small intestine, PPs are oval and irregularly distributed along the antimesenteric side of the gut [2]. At the opposite, in the distal ileum, they are numerous and they form a lymphoid ring [4] (Figure 1). Indeed, at least 46% of PPs are concentrated in the distal 25 cm of ileum in Human [4]. It is to note that there are large variations in size, shape, and distribution of PPs from one individual to another one. The consequences of these variations on the physiological and/or pathological parameters related to PP functions remains to be elucidated [2, 4].

### 2.1. Development of Peyer's Patches

**In Human**

The fetal human small intestine contains in average 60 PPs before week 30 of gestation and their number steadily increase reaching a maximum of 240 at puberty [2]. *Baginskys* and others identified distinct clusters of T and B cells in the small intestine at 14–16 weeks of gestation [2, 5–8]. At week 19, these aggregates mature into recognizable PPs containing follicular dendritic cells (FDCs) and become macroscopically discernable at week 24, even though no germinal centers are present. The latter rapidly develop after birth, when the intestines are exposed to commensal bacteria and antigens [2]. Although macroscopic descriptions of human PP are available, no information concerning the embryonic steps of PP development is actually reported whereas the different steps of PP genesis have extensively been studied in mice.

**In Mouse**

Three successive steps have been evidenced in PP formation in mouse. The first one, at embryonic day 15.5 (E15.5), marks the beginning of PP development. At that time, VCAM-1 is expressed by distinct clusters of stromal cells located on the antimesenteric side of the small intestine [9]. These VCAM-1 positive cells also express the ligand of the tyrosine kinase receptor RET [10]. During the second step (between E15.5 and E17.5), VCAM-1 positive cells recruit RET^+^CD11c^+^cKit^+^lymphotoxin^+^ cells and IL7R^+^lymphotoxin^+^CD4^+^CD3^−^ LTic (Lymphoid Tissue inducer cells) [9–11]. The VCAM-1-positive stromal cells express the lymphotoxin *β* (LT*β*) receptor, and upon ligation of this receptor produce IL7 and homeostatic chemokines such as CXCL13 [12]. This reciprocally leads to increased expression of surface lymphotoxin on LTic, forming a self-sustaining PP primordium [13, 14]. Gene inactivation of CXCL13 and LT*β*-receptor interrupts the interaction of LTic with organizer cells and thus abolishes PPs development. Similarly, injection of LT*β*R fuses to a truncated human immunoglobulin competitively interferes with LT*β*R signaling by organizer cells and interferes with PP development. Since E17.5, during the third phase of PP genesis, circulating lymphocytes are attracted. They enter into the developing organs and fill up the T and B cell niches [11]. While the embryonic genesis of PPs is largely known, their postnatal development is actually poorly understood (see Section 4.1).

### 2.2. Architecture of Peyer's Patches

**Figure 2:**
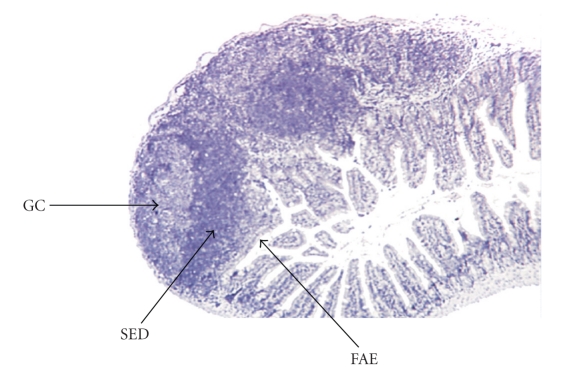
Histological features of a Peyer's patch. Three follicles are individualized. Arrows show the germinal center (GC); subepithelial dome (SED) and follicle associated epithelium (FAE) for one of these follicles.

Morphologically, PPs are separated into three main domains: the follicular area, the interfollicular area and the follicle-associated epithelium [1]. The follicular and interfollicular areas consist of the PP lymphoid follicles with a germinal center (GC) containing proliferating B-lymphocytes, follicular dendritic cells (FDCs) and macrophages. The follicle is surrounded by the corona, or subepithelial dome (SED) containing mixed-cells including B-cells, T-cells, macrophages and dendritic cells (DCs). PPs are connected to the body by lymphatic vessels and endothelial venules. Naïve lymphocytes immigrate into the PP via specialized high endothelial venules. Naïve or active lymphocytes leave the PP via efferent lymphatic vessels at the serosal side of the PPs which connect the PPs to the mesenteric lymph nodes (MLN). The arched appearance of PPs is due to the GC forming the core of each follicle (Figure 2).

The follicle-associated epithelium (FAE) differs from the epithelium of the villus mucosa: the production of mucus is weak; the membrane-bound digestive enzymes are lightly expressed and the enterocyte brush border glycocalyx has different glycosylation patterns [15–17]. FAE is also characterized by a large number of infiltrated B-cells, T-cells, macrophages and DCs. Finally, the FAE lacks the subepithelial myofibroblast sheath and, the basal lamina is more porous compared with the regular epithelium [18, 19].

FAE are constantly renewed from precursor cells located in adjacent crypt zones [20]. The main feature of FAE is the presence of M-cells which are specialized enterocytes. M-cells differentiate from enterocytes under the influence of membrane-bound lymphotoxin (LT*α*1*β*2) present on local lymphoid cells, mainly B-cells [21]. The cellular composition of the FAE (i.e., the proportion of enterocytes and M-cells) may be modulated by bacteria present in the gut lumen. For example, the number of M-cells in FAE is increased after transfer of mice from pathogen-free to normal housing conditions [22]. Pathogenic bacteria like *Streptococcus pneumoniae *or* Salmonella typhimurium* may increase the number of M-cells within the FAE [23, 24]. Thus the FAE exhibit an astonishing phenotypical plasticity and can rapidly change its functions depending on host or bacterial stimuli.

M-cells are specialized in the transcytosis of intact luminal material like soluble proteins, antigens, bacteria and viruses [25]. Endocytosis, phagocytosis, pinocytosis, and macropinocytosis are all mechanisms used for the ingestion of the extracellular material. M-cells highly express diverse glyco-signatures which may be exploited as receptors by some microbes [25]. They also express IgA receptors allowing the capture and uptake of IgA trapped bacteria [26]. As a result, luminal IgA not only prevents penetration of bacteria/pathogen into the mucosa but also redirects them to the M cells and PPs [27].

The paracellular permeability is differentially regulated into the FAE [28, 29]. Compared with intestinal mucosa, the FAE exhibits an increased expression of claudin-3 and occludin, which are both described to downregulate the opening of tight junctions. [28]. On the contrary, claudin-2 (which is known to have an opposite effect), is less expressed in FAE than in the villus epithelium [28]. The site of expression may vary within the FAE: claudin-3 and occludin are expressed throughout the dome whereas claudin-4 is preferentially seen in the apex region of the dome [28] and Claudin-2 in the boarding villus epithelium [28]. Moreover, Clark and Hirst found that the adherens junctions of murine M-cells could be recognized by enhanced expression of *β*-catenin, *α*-actinin, and polymerized actin [29].

### 2.3. Cellular Composition of Peyer's Patches

**In Human**

Because it is difficult to identify and collect PPs during routine endoscopies, studies of human mucosal lymphoid follicles are rare and limited to young patients. In human, among the mononuclear cells (MC), CD4^+^/CD25^+^ (10%) cells and CD8^+^/CD25^+^ (5%) cells are more abundant in PPs than in the peripheral blood [30]. Nagata et al. observed that after incubation with *β*-lactoglobulin, CD4^+^ and CD8^+^ T-cells from PPs were orientated toward a Th1 profile (characterized by the production of IFN*γ*) but not toward a Th2 profile (characterized by IL-4 secretion) [30]. Junker et al. investigated the cellular subsets within the isolated lymphoid formations (ILFs) [31]. T-cells were found more frequently CD4^+^ and CD62L^+^ than CD8^+^ and CD103^+^ cells [31]. In addition, antiCD3/CD28 stimulation induced a proliferation of T-cells associated with the secretion of high levels of IFN*γ*, TNF*α* and interleukin (IL)-2, but low levels of IL-4, IL-6 and IL-10 [31], confirming that PPs present a Th1 rather than a Th2 profile. Whereas very few papers report human PP's cellular composition, mouse PPs have extensively been studied.

**In Mouse**

PP exhibit about 60% of B-cells (B220^+^), 25% of T-cells (CD3^+^), 10% of dendritic cells (CD11c^+^) and less than 5% of macrophages (F4/80^+^) or polymorphonuclear neutrophil (Ly-6G^+^). Among T-cells, 45% are CD4^+^, 35% are CD8^+^ and 20% are CD4^−^/CD8^−^ T-cell. Among CD4^+^ T cells, 85% are memory T-cells (CD25^−^CD45RB^lo^), 10% are Naive (CD25^−^CD45RB^hi^) and 5% are regulatory T-cells (CD25^+^CD45RB^lo^) [32]. Distinct subsets of DCs, based on their cell-surface marker expression, together with their location, have been identified in PP [33, 34]. All the subsets express CD11c and major histocompatibility complex class II antigens but differ for their expression of CD8*α* (lymphoid) and CD11b (myeloid) molecules. Lymphoid CD11c^+^CD8*α* ^+^CD11b^−^ DCs are localized within the T-cell–rich interfollicular regions [33]. Myeloid CD11c^+^CD8*α* ^−^CD11b^+^ DCs are present under the FAE in the SED [33]. Finally, the “double negative” CD8*α* ^−^CD11b^−^ DCs are found in the SED, the interfollicular region, and within the FAE [33].

In comparison with DCs from spleen (SP), DCs derived from PPs exhibit strong functional differences [35]. PP DCs are more potent in stimulating allogeneic T-cells proliferation compared with DCs from SP, and DCs derived from PPs, but not from SP, are able to prime the production of IL-4 and IL-10 (Th2 anti-inflammatory cytokines) [35]. In addition, PP DCs prime T-cells for the production of much lower levels of IFN*γ* (Th1 inflammatory cytokine) compared with SP DCs. Finally, stimulation of PP DCs with CD40 molecule resulted in secretion of high levels of IL-10, whereas the same stimulus induced no IL-10 secretion from SP DCs. All DC subpopulations derived from PP secrete a distinct pattern of cytokines upon exposure to T-cells and microbial stimuli. CD8*α* ^+^CD11b^−^ (lymphoid) and double negative DCs share similar functional characteristics as they both orientate the T-cells toward a Th1 profile, notably via IL-12 secretion upon bacterial stimulation [34]. In contrast, only CD8*α* ^−^CD11b^+^ myeloid DCs produce high levels of IL-10 upon stimulation with CD40 ligand, or *Staphylococcus aureus*. In addition, myeloid DCs are particularly capable of priming naive T cells to secrete high levels of IL-4 and IL-10 (Th2 anti-inflammatory cytokines), when compared with those from extramucosal sites, while lymphoid and double negative DCs from all tissues prime for IFN*γ* (Th1 inflammatory cytokine) production [34].

Very recently, a new subset of myeloid dendritic cells (CD11c^+^CD11b^+^) has been identified in the subepithelial dome of mouse and human PP. These DCs strongly express lysozyme and are able to internalize bacteria and dead cells. Moreover these DCs possess the machinery required to efficiently present antigens to the immune cells—class II major histocompatibility complex and costimulatory molecules—thus actively participating in the first immune defense line within PPs [36].

### 2.4. Involvement of Peyer's Patches in the Induction of Oral Immune Tolerance

 The function of PPs was unknown until 1922, when Kenzaburo Kumagai reported an uptake of *Mycobacterium tuberculosis* inside the epithelial dome of PP. However, as he also observed an uptake of heat-killed bacteria and sheep red blood cells by PPs, he concluded that this uptake was a nonspecific process. Nevertheless, Owen and Jones showed in 1974 that M-cells were able to take up antigens highlighting the role of PPs in the immune system [15].

Immunological tolerance against non-pathogenic bacteria and antigens is a phenomenon observed along the gastrointestinal mucosa [37] which avoids reactions against proteins and commensal bacteria. Oral tolerance is an active process, leading to the generation of antigen-specific T lymphocytes that suppress further immune stimulation. It is defined by the antigen-specific suppression of both cellular and humoral immune responses to orally administered antigens. In addition to the generation of suppressive T cells, anergy and T cell deletion have been described as mechanisms underlying oral tolerance [38]. Consequently, mucosal tolerance protects the mucosa from detrimental inflammatory immune responses. The activation involved in the tolerance induction process to proteins is also important for the maturation of the immune system. As an example, mice feed with a protein-free diet exhibit an underdeveloped GALT with low amounts of immunoglobulin A together with a systemic Th2 profile [39]. A defect in the generation of suppressive T-cells against food or commensal bacterial antigens could lead to food hypersensitivity and celiac disease [40, 41]. Consequently, mucosal tolerance protects the mucosa from detrimental inflammatory immune responses. 

Oral tolerance to a broad variety of antigens involves the suppression of different types of immune responses, including delayed hypersensitivity and antibody production. PPs have been extensively studied for their contribution to mucosal tolerance, but their precise role is still unclear. After oral administration of antigens, PPs are the first places of T-cell-specific priming and proliferation in the gut [42]. Mice lacking PPs fail to generate an oral tolerance against ovalbumin but develop an oral tolerance toward small chemical haptens like TNBS suggesting that organized PPs are involved in protein unresponsiveness while epithelial cells modulates the response to smaller molecules [43]. However other observations suggest that this point of view may be too simple: surgical removal of PPs does not interfere with the ability of rats to develop an oral tolerance [44]; an oral tolerance toward proteins has been reported in mice lacking PPs in specific conditions [45, 46]; and the administration of antigens in isolated intestinal loops with or without PPs induced a tolerance in both conditions [47]. Noteworthy, gradual decline in PP immunological functions has been implicated in the lack of oral tolerance in aging mice [48]. Thus, if PPs are clearly very efficient in the uptake and handling of antigens, their exact role in the induction of oral tolerance remains to be clarified.

### 2.5. Role of Peyer's Patches in the Defense against Pathogens

 As previously described, the FAE and M-cell phenotypes are optimized for antigen and microorganism uptake and handling. The mechanisms by which M-cells take up microorganisms and macromolecules vary according to the nature of the biological material. Large particles and bacteria induce phagocytosis, which is often associated with ruffling of the apical plasma membrane of the M cell and rearrangement of the actin cytoskeleton, which permits active formation of pseudopodia-like structures [49, 50]. Viruses and other adherent particles are taken up by endocytosis via clathrin-coated vesicles, whereas non-adherent material is internalized by fluid phase endocytosis [27, 51, 52]. In all these cases, internalization is followed quickly by transport of endocytotic vesicles to the endosomal compartment and then by exocytosis to the basolateral membrane. PP sampling of the lumen is crucial for protective mucosal immune responses. As a counterpart, PPs provide a route of entry into the organism for various pathogenic agents such as bacteria, viruses, protozoa or prion.

**Bacteria**

Among the pathogenic bacteria with a digestive tropism such as *Escherichia coli*, *Yersinia, Mycobacterium avium paratuberculosis*, *Listeria monocytogenes*, *Salmonella typhimurium *and, *Shigella flexneri*, all of them have been reported to invade the host by adhering with FAE M-cells.

Most of the strains of *E. coli* do not adhere to M-cells but the *Enterohaemorrhagic E. coli* (EHEC) and *enteropathogenic E. coli* (EPEC) show specific adherence to FAE when cocultured with human intestinal biopsies [53, 54]. Infection with the EHEC strain O157:H7 causes diarrhea, hemorrhagic colitis and hemolytic uremic syndrome [55]. This strain selectively adheres to FAE by its intimin-*γ* protein and binds the *β*1-integrins expressed on the M-cell apical surface [56, 57]. Other enteropathogenic *E. Coli* strains (like EPEC RDEC-1) adhere to the M-cells but with a mechanism independent of intimin [58, 59]. Finally, some EPEC strains like O127:H7 exhibit a similar rate of translocation across M-cells and enterocytes *in vitro* [60]. In addition, it was observed that translocation rates were significantly increased in the absence of a functioning Type III secretion system [60].

*Yersinia enterocolitica and Y. pseudotuberculosis* are human foodborne pathogens that cause clinical ileitis or ileocolitis. *Yersinia species* adhere to both enterocytes and M-cells but with a preference for M-cells [61–63]. *Y. enterocolitica* and *Y. pseudotuberculosis* targets the M-cells via the molecular interaction between the *β*1 integrins present on the host cell and invasin, an outer-membrane *Yersinia* protein [61, 62, 64]. As a result, Yersina causes major damages to PPs and bacterial mutants lacking the invasin protein display reduced colonization and translocation of PPs *in vivo* [57, 62].

Paratuberculosis or Johne's disease is a chronic enteritis of the cattle and other small ruminant caused by *Mycobacterium avium paratuberculosis (MAP)*. In human, MAP ingestion causes acute and chronic enteritis. MAP are able to invade the intestinal mucosa by interacting with enterocytes [65] and M-cells [66, 67]. *In vitro* studies have shown that the attachment and the internalization of *MAP* by epithelial cells depend on the interaction between Fibronectin attachment proteins and fibronectin [68–70]. In fact *β*1 integrins are the host cell receptors for fibronectin-opsonized mycobacteria [68, 71]. Because M-cells are the unique intestinal cells expressing *β*1 integrins at a high density on their luminal surface, they represent the main entrance site for *MAP* [64].

*Listeria monocytogenes* is the causative agent of human listeriosis, a potentially fatal foodborne infection. Clinical manifestations range from febrile gastroenteritis to more severe invasive forms, including sepsis, meningitis and rhombencephalitis. *L. monocytogenes* invades nonphagocytic cells such as enterocytes and this process is critical for bacterial translocation through the intestinal epithelium [72, 73]. While it is clear that the pathogen interacts with the enterocytes via internalins, several observations suggest that *L. monocytogenes* has also the potential to invade their host via M-cells. First, a rapid localization of *L. monocytogenes* into mouse PPs has been reported [74, 75]. Second, *L. monocytognenes* migrates through differentiated M-cells more efficiently than in non differentiated cells *in vitro* [75]. Finally, *in vivo* analysis of orogastric *L. monocytogenes *infections showed a preferential replication within the PPs with an extremely rapid translocation to internal organs [76, 77]. Moreover, it has been shown that *L. monocytognenes* migrates through differentiated M-cells more efficiently than in non differentiated M-cells [75].

In contrast with *Mycobacterium *[49] or* Yersinia [61, 78]*, which have been shown to specifically attach to and pass through M-cells without modifications or died M-cells, *Shigella flexneri *[79] and *Salmonella typhimurium* [80, 81] are known to alter M-cell homeostasis and functions. *Shigella flexneri* requires both an adhesive and invasive phenotype to efficiently colonize FAE. Following *Shigella *infection, M cells begin to increase in size, which eventually disrupts the integrity of the epithelium [79]. The effect of invasive *Salmonella typhimurium *on M-cells is dramatic [80, 81]. At the earliest stages of *Salmonella *invasion, large membrane ruffles appear on the apical surface of the M-cells, and within a short period of time (30 to 60 min), the cells becomes necrotic and begins to die. Finally, although PPs have been recently involved in the *Helicobacter pylori* induced gastritis, it has been observed that the translocation of *H. pylori* across PPs is performed by DC [82]. Thus, no gastritis is induced in *H. pylori*-infected mice lacking PPs and it has been evidenced that the coccoid form of *H. pylori is* phagocytosed by DC in PPs [82].

Together, these findings indicate that enteric pathogens have evolved distinct mechanisms to interact, invade and destroy PPs. Although the majority of enteric bacteria alter PP homeostasis by interacting and invading M cells from FAE, DCs inside FAE seem to play an alternative pathway.

**Viruses**

Several viruses like Reovirus type-1, Poliovirus and HIV type 1 are transported by M-cells [83–85].

*Reovirus* is an orally transmitted murine pathogen, which affects the nervous system, causing encephalitis. Reovirus type-1 selectively adheres to M-cells by interacting with *α*-2-3-linked sialic acid glycoconjugates expressed by M-cell [86]. The infection causes a depletion of the M-cells from the FAE [87].

*Poliovirus* is the causative agent of poliomyelitis. It infects humans via the oral route. PPs are the primary sites of virus replication in the gut [83]. In human infected tissues, virions were specifically found on the surface and in intracellular vesicles of M-cells [83].

Transmission of HIV type 1 (HIV-1) infection via anorectal, cervicovaginal, foreskin and urethral epithelia accounts for 80% of AIDS cases [84]. HIV-1 is able to cross the mucosal barrier of the intestinal or genital tracts to infect CD4^+^ T-cells. HIV-1 can adhere to M-cells—via the chemokine receptor CXCR4 expressed apically on M-cells [88] but not to enterocytes [84].

**Prion**

Transmissible spongiform encephalopathies (TSE) are characterized by the accumulation of a protease-resistant abnormal isoform of the prion protein (PrP^Sc^), which is converted from the cellular isoform of the prion protein (PrP^c^). After oral transmission, PrP^Sc^ can invade the host through PPs [89–91]. In mouse models, reduced PP numbers have been associated with a higher resistance to orally acquired prion infection [91]. Moreover, it has been suggested that the prion protein migration from the gut to the lymphoid system also involve M-cells [92]. Finally, the replication and the accumulation of prion during TSE seem to be located in the FDCs of PP. Altogether, these studies argue for a major role of PPs in TSE pathogenesis [93–95].

## 3. Peyer's Patches: A Key Organ of the Relationship between Innate and Adaptative Immunity in the Gut

### 3.1. The Nod2 Sensor in Peyer's Patches

Pathogen associated molecular patterns (PAMPs) present on commensal and pathogenic bacteria are recognized by pathogen recognition receptors (PPRs) present in the host cells. Among the PAMPs, the Toll like receptors (TLRs) and the Nucleotide oligomerisation domain (NODs) are largely expressed in follicle associated cells such as epithelial or dendritic cells. TLRs are mainly extracellular sensors whereas the Nods are cytoplasmic. TLRs and Nods are triggered by a different set of PAMPs. Particularly, Nod2 is able to recognize the muramyl dipeptide (MDP) a component of the peptidoglycan bacterial wall present in most Gram^+^ and Gram^−^ bacteria. Common *NOD2* variants have been associated with Crohn's Disease (CD) [96, 97] and graft-versus-host disease (GVHD) [98, 99]. The main CD and GVHD associated variants—R702W, G908R and 1007fs—are located within or near the Leucin rich repeat domain (LRR) that is supposed to interact with the MDP [98–100]. 

While lymphotoxin and IL-7 signalling are essential for the organogenesis of PP during the embryonic stage, studies on germ-free animals argue for a critical role of the gut flora during postnatal development [1]. Germ-free animals have an underdeveloped GALT and are resistant to experimental colitis and to severe GVHD [101], suggesting that bacterial sensors could be implicated in PP development and the Human diseases. Whereas, it has been evidenced a reduction of PP size in TLR deficient mice [102], invalidated mice for *Nod2* gene (*Nod2^−/−^*) are characterized by a hypertrophy and a hyperplasia of the GALT [32, 103]. After birth NOD2^mut/mut^ mice carrying a frameshift mutation homologous to the Human 1007fs variant exhibit a phenotype comparable to that of *Nod2^−/−^* mice [103].

In fact, *Nod2* seems to play a pivotal role in the GALT homeostasis in response to commensal bacteria [104]. The expression of *Nod2* depends on the presence of commensal bacteria: while its expression in the terminal ileum of mice rederived into germ-free conditions decreased significantly, it is induced by commensal bacteria into germ-free mice [104]. In addition, chronic antibiotic therapy abrogates the overdevelopment of the GALT in *Nod2^−/−^* mice [103]. Gut microflora exerts a strong stimulation on the *Nod2^−/−^* PPs mice, inducing a high proportion of CD4^+^ T-cells, high levels of inflammatory cytokines and high permeability rates for antigens and bacteria [103]. In turn, the terminal ileum of *Nod2^-/^* ^−^ mice exhibits an elevated load of commensal bacteria and its ability to prevent intestinal pathogenic bacteria colonization is diminished [104]. As a result, Nod2 appears to play a key role in the regulation of the interaction between PP and the gut flora.

### 3.2. Nod2: A Link between Innate Immunity and Adaptative Immunity

*Nod2* appears not only to influence the development of the GALT but it is also able to modulate the immune response toward bacteria, by limiting the development of a Th1 immune response. In wild type mice DCs, MDP acts synergistically with lipopolysaccharid (LPS)—the TLR4 ligand—to promote the proliferation of naïve CD4^+^ T-cells with a Th2-like cytokine profile. By contrast, DCs carrying *Nod2* mutations are unable to react to MDP, but respond to LPS and promote the development of Th1-orientated cells [105]. As a result, *Nod2* seems to limit the ability of DCs to induce a polarised Th1 response of CD4^+^ T-cells [105]. Similar data have been evidenced in mice, where Nod2 stimulation by MDP triggers a potent age-specific immune response with a Th2-type polarization profile, characterized by the induction of IL-4 and IL-5 by T cells and IgG1 antibody responses [106]. Nod2 was also found to be critical for the induction of both Th1- and Th2-type responses following costimulation with TLR agonists [106]. Because this synergistic response was recapitulated by DC *in vitro*, it can be supposed that DCs likely play a central role in the integration of Nod2- and TLR-dependent signals for driving the adaptive immune response [106]. Together, these data identify Nod2 as a critical mediator of microbial-induced potentiation and polarization of age-dependent immunity. 

In the absence of *Nod2*, PPs present a higher rate of CD4^+^ T-cells and M-cells in the FAE and increased levels of Th1 (IFN*γ*, TNF*α* and IL-12) and Th2 (IL-4) cytokines. These immune alterations are associated with an increased of paracellular permeability and yeast/bacterial translocation [32]. Indeed, PPs from *Nod2* ^−/−^ mice exhibit an elevated translocation of *Escherichia coli*, *Staphylococcus aureus* and, *Saccharomyces cerevisiae* [32]. This increase of microbes passage is mediated by an upregulation of myosin light chain kinase expression and activity [103]. CD4^+^ T-cell depletion and IFN*γ*-blocking antibodies in *Nod2* deficient mice abrogated this phenotype [103]. Altogether, these data suggest that *Nod2* modulates the adaptive immune response of PPs and may promote the immune tolerance. As a result, Nod2 also regulates the intestinal barrier function, limiting the paracellular and transcellular permeabilities together with bacterial translocation.

Altogether, these data support the contribution of *Nod2* in the immunogenic tolerance toward gut microflora and a key role of *Nod2 *in CD4-T cells function. Studies focusing on GVHD also argue for the capacity of Nod2 to regulate the T-cell response. GVHD is a common complication of allogeneic stem cell transplantation, which occurs when donor-derived T-cells are stimulated by host antigen-presenting cells. Acute GVHD is characterized by damages mainly in the skin, the liver, the gastrointestinal tract and other mucosae. Using an experimental model of *Nod2* chimeric mice, *Penack* and coworkers have shown an exacerbated GVHD in case of allogenic transplantation of Nod2^+/+^ mice with *Nod2^−/−^* bone marrow cells [107]. As expected, this phenotype was associated with an increased activation and proliferation of alloreactive donor T-cells and* Nod2* deficient DCs were involved in the phenotype [107]. At the opposite, allogenic transplantation of *Nod2^−/−^* mice with Nod2^+/+^ bone marrow cells had no significant impact on the development of GVHD [107]. However, this important role of Nod2 in the T cell function does not seem to be confirmed in human. In human, GVHD proceeded by an allogenic stem cell graft and immunosuppressive prophylaxis, the analysis of biopsies from intestinal GVHD showed a decrease of CD4^+^-T cells infiltrate when recipient carried *NOD2* GVHD associated variants whereas the donor *NOD2* status had no significant impact on the CD4^+^ cell infiltrates [108].

Nod2 also plays a role in the immune response to pathogens. For example, *Nod2* deficient mice are more susceptible to *Toxoplasma gondii* infection [109]. This observation was associated with a defect of IFN*γ* production by Th1 lymphocytes. Interestingly, this phenotype was not due to a lack of CD4^+^ T-cell activation by DCs. In a model of *Mycobacterium tuberculosis* infection, Divangahi et al., showed that *Nod2* deficient mice exhibited a decreased production of Th1 cytokines—IFN*γ* and TNF*α*—as well as a reduced recruitment of CD4^+^ and CD8^+^ T cells [110]. 

If Nod2 modulates the adaptive immune response, its mechanisms of action are probably multiple. In human monocytes-derived DCs *Nod2* is able to induce the autophagy after activation by the MDP. By consequence, it promotes bacterial handling and activates the major histocompatibility complex class II antigen-specific CD4^+^ T cell responses [111]. Nod2 activation also enhances the TLR-dependent induction of IL-1 and IL-23, thus promoting Th17 orientated T-cells which have been implicated in antimicrobial response [112]. Finally, the study of Shaw et al. argues for a proper role of *Nod2* in T-cell function independently of DCs and MDP induction. In their model of *T.gondii *infection, DCs from *Nod2* deficient mice were able to activate a normal response of wild type T-CD4^+^ to *T.gondii* suggesting an intrinsic role of *Nod2* in the generation of an effective Th1 response [109]. Moreover Rick was not necessary to protect against *T.gondii *suggesting the implication of a pathway independent of the *Nod2*-MDP-activation in CD4^+^ T-cells [109]. Similarly, it has been recently evidenced that NOD2 exerts an important role in the human regulatory T-cells (Treg cells): NOD2 stimulation results in the upregulation of antiapoptotic genes in human Treg cells [113]. In addition, Crohn's disease *NOD2* variants are associated with a deficiency of FOXP3^+^ Treg cells in the colonic lamina propria [113].

Although the different mechanisms by which *Nod2* promote T-cell response are not fully understood it appears now clearly that *Nod2* has a role not only in innate immunity but also in adaptive immunity.

## 4. Peyer's Patches and Human Diseases

### 4.1. Crohn's Disease

Crohn's disease (CD) is an inflammatory disorder characterized by a chronic or relapsing inflammation of the digestive tract. A key role of PPs in CD has been supported by a spatiotemporal relationship between the CD lesions and PPs and by the pathogenesis on CD which is supposed to result of an inappropriate innate and/or adaptative immune response to the bacterial flora. 

CD can affect all the digestive tract areas with a preference to the terminal part of the ileum where PPs are more numerous [4]. The number and size of PPs increase from birth to 15–25 years old and then decline with age. This curve is roughly parallel with the age-incidence curve of CD, [114] this is especially true for the ileal presentation of the disease considering that ileal CD is rare in young children and seniors [115, 116]. These observations argue for a temporal relationship between PP development and CD as proposed by Van Kruiningen et al. [2, 4, 115]. Finally, the very early CD lesion, a tiny ulcer called aphtoid lesion has been found by several authors to be centered by lymphoid follicle formations [117–119]. In carefully performed correlative studies with magnifying endoscopy and scanning electron microscopy, *Fujimura* and coworkers demonstrated that the aphtoid lesions of CD are preceded by ultrastructural erosions (150–200 microns in size) in the FAE of hyperemic lymphoid follicles [4, 120].

It is largely admitted that CD is associated with an abnormal T-cell-mediated immune response toward the gut flora. Inflammatory lesions of CD (i.e., aphtoid lesion and ulcers) are more pronounced in the terminal ileum and colon which contain the highest densities of bacteria. The partial efficacy of antibiotics and fecal diversion in CD patients also highlight the fundamental role of bacteria in CD pathogenesis. Now, several genes implicated in bacterial recognition and/or innate immunity including *NOD2* but also the autophagic genes *ATG16L1* and *IRGM *have been implicated in genetic CD susceptibility. Actually, studies on CD microbiota have found evidence for decline in bacterial diversity in CD patients, compared to controls [121, 122]. Because PPs are specialized in sampling and presenting luminal antigens and bacteria to the underlying immune cells, a few authors have studied the role of PPs in CD pathogenesis. Keita et al. have shown an increased translocation of non pathogenic *E. Coli* associated with an increased percentage of *E. Coli* colocalizing with DCs in PPs of ileal CD compared to controls [123]. More recently, these DCs have been characterized by FACS analysis and immunofluorescence microscopy, leading to the identification of a subset of mature CD83^+^CCR7^−^ DC, able to internalize live bacteria [124]. 

PPs have a pivotal role in the interaction between gut bacterial flora and immune response/tolerance. Their participation in digestive inflammatory disorders such as CD and their interplay with the function and diversity of the gut microbiota is becoming a productive field of research.

### 4.2. Graft versus Host Disease

Like for CD, in acute GVHD, the interplay between the bacterial flora and the epithelial immune response contributes to inflammatory signals that enhance the donor-derived T cells stimulation by host antigen-presenting cells. The first evidence of the role of GALT in GVHD was provided by *Bekkum* and coworkers in 1974 when they reported that germ-free mice were resistant to enteric GVHD in a model of irradiation followed by incompatible bone marrow transplantation. Using a model of acute GVHD in PP-deficient mice, Murai et al. demonstrated that PPs are the anatomical site for the infiltration of donor CD8^+^ T-cells and generation of antihost cytotoxic T-cells [101]. However, other authors reported that PPs are not required for the induction of acute GVHD when myeloablative conditioning is applied before bone marrow transplantation [125]. Thus, even if the implication of PP in the pathogenesis of acute GVHD is still in debate, PPs that are at the interface between bacterial flora and immune response have a pivotal role in alloresponse and inflammation.

## 5. Conclusive Remarks

PPs are key players of the mucosal immune host response toward gut antigens and bacteria. Their function remains to be clarified in many aspects including the regulation of T-cell differentiation after antigen exposure. Nod2 seems to play a crucial role at the interface between innate and adaptive immunity in PPs. It is involved in PP development in response to the commensal flora. It also plays a role in PP permeability, translocation and response toward pathogenic bacteria which exploit PP for their virulence. These findings may be helpful to better understand the mechanisms involved in *NOD2* associated diseases like CD and GVHD.

## Abbreviations

CD: Crohn's disease
EHEC: Enterohaemorrhagic *Escherichia coli *
EPEC: *Enteropathogenic Escherichia coli*
FAE: Follicle-associated epithelium
FDCs: Follicular dendritic cells
GALT: Gut-associated lymphoid tissue
GC: Germinal center
GVHD: Graft versus host disease
ILFs: Isolated lymphoid formations
LPS: Lipopolysaccharid
LT: Lymphotoxin
LTic: Lymphoid Tissue inducer cells
M cell: Microfold cell
MDP: Muramyl dipeptide
MLN: Mesenteric lymph nodes
NODs: Nucleotide oligomerisation domains
PAMPs: Pathogen associated molecular patterns
PBMC: Mononuclear cell from peripheral blood
PPs: Peyer's patches
PPMC: Mononuclear cell from PP
PPRs: Pathogen recognition receptors
PrPC: Cellular isoform of the prion protein
PrP^Sc^: Protease-resistant abnormal isoform of the prion protein
SED: Subepithelial dome
TLRs: Toll like receptors
TSE: Transmissible spongiform encephalopathies.
